# Performance Characteristics of Real-Time PCRs for African Swine Fever Virus Genome Detection—Comparison of Twelve Kits to an OIE-Recommended Method

**DOI:** 10.3390/v14020220

**Published:** 2022-01-24

**Authors:** Jutta Pikalo, Tessa Carrau, Paul Deutschmann, Melina Fischer, Kore Schlottau, Martin Beer, Sandra Blome

**Affiliations:** Friedrich-Loeffler-Institut, Suedufer 10, 17493 Greifswald-Insel Riems, Germany; Jutta.Pikalo@gmx.at (J.P.); Tessa.CarrauGarreta@fli.de (T.C.); Paul.Deutschmann@fli.de (P.D.); Melina.Fischer@fli.de (M.F.); Kore.Schlottau@fli.de (K.S.); Martin.Beer@fli.de (M.B.)

**Keywords:** African swine fever virus, laboratory diagnosis, commercial real-time PCR, performance, sensitivity, specificity

## Abstract

African swine fever (ASF) is a major threat to pig production, and real-time PCR (qPCR) protocols are an integral part of ASF laboratory diagnosis. With the pandemic spread of ASF, commercial kits have risen on the market. In Germany, the kits have to go through an approval process and thus, general validation can be assumed. However, they have never been compared to each other. In this study, 12 commercial PCR kits were compared to an OIE-recommended method. Samples representing different matrices, genome loads, and genotypes were included in a panel that was tested under diagnostic conditions. The comparison included user-friendliness, internal controls, and the time required. All qPCRs were able to detect ASFV genome in different matrices across all genotypes and disease courses. With one exception, there were no significant differences when comparing the overall mean. The overall specificity was 100% (95% CI 87.66–100), and the sensitivity was between 95% and 100% (95% CI 91.11–100). As can be expected, variability concerned samples with low genome load. To conclude, all tests were fit for purpose. The test system can therefore be chosen based on compatibility and prioritization of the internal control system.

## 1. Introduction

African swine fever virus (ASFV) is a large, enveloped, double-stranded DNA virus. Its genome comprises 170–190 kbp with up to 167 open reading frames [1,2]. This virus is the only member of the *Asfarviridae* family and the only known DNA arbovirus affecting vertebrates [1,2]. It is the causative agent of the highly lethal hemorrhagic disease African swine fever (ASF), that affects *Suidae* species of all breeds and ages. Outbreaks of ASF result in severe socioeconomic disturbances worldwide, and can endanger livelihoods under rural settings [3]. For this reason, the disease is notifiable to the World Organization for Animal Health (OIE). Up to now, 24 genotypes have been described based on the variation of the p72 capsid protein, encoded by the B464L gene located in the C-terminal region of the genome [4,5,6,7,8].

After its introduction to the European continent in 2007 via Georgia, the disease spread north- and west-wards, reaching Germany in 2020 [3,9]. Beyond, the virus also reached Asia in 2018, and Hispaniola in 2021 (OIE WAHIS, visited online on 18 December 2021). The ASFV strains involved in this pandemic belong to the p72 genotype II and show high virulence in the vast majority of cases, inducing an acute form of ASF [3].

Despite the severe clinical presentation, most clinical signs are unspecific, and laboratory diagnosis is mandatory to confirm any clinical suspicion [10]. In this context, the real-time polymerase chain reaction (qPCR) provides a sensitive, specific and fast tool for the detection of ASFV [11]. Several qPCR assays have been described in literature for detection of ASFV and the reference laboratories follow the recommendation given by the OIE or regional reference laboratories, e.g., the European Union Reference Laboratory for ASF [11,12,13,14]. However, over the last few years and because of the daily new ASFV outbreaks, commercially available kits have risen on the market, both on European level and globally. Within the EU, the national reference laboratories (NRLs) have to ensure the quality of the diagnostic tests and their adherence to international standards. Should those commercial in vitro diagnostic kits be used in Germany in particular, approval is needed by the German Licensing Authority. Under these circumstances, it can be assumed that the certified products are validated and fit for purpose in general. However, a continuous and independent assessment of these products is necessary to guide the implementation of accurate tests in the ASFV diagnostic market, and to ensure that stakeholders can make informed decisions.

The aim of this study was to compare and analyse the performance of these commercially available and mostly approved qPCR kits with an assay recommended by the OIE [11]. In addition to the reliable amplification of the target sequence, the main focus of this study, the comparison also included user-friendliness, the robustness of the internal controls and the time required. To this end, a panel reflecting different sample matrices, ASFV genome loads, and genotypes was employed in a diagnostic manner, i.e., in a single-run approach with manual evaluation of amplification curves, thresholds, and plausibility check.

## 2. Materials and Methods

### 2.1. Samples

The selection of specimens was made according to the following parameters: (i) infection status of the host, (ii) disease course and genome load, (iii) ASFV p72 genotype, (iv) sample matrix and (v) host species. To this end, a total of 207 samples (*n* = 179 ASFV-positive; *n* = 28 ASFV-negative), representing 18 p72 genotypes of ASFV were chosen. The matrices included blood, different organs, swabs, and cell culture supernatants. The diagnostic status of all samples was established prior to carrying out the study at the accredited German NRL for ASF by using the methodology described elsewhere [9]. The detailed description of each sample is listed in the Supplementary Material Table S1.

#### 2.1.1. Field Samples

One part of the samples (*n* = 80; *n* = 73 ASFV-positive; *n* = 7 ASFV-negative), named “*field samples*”, were specimens submitted by the local authorities to the German NRL during the ASFV outbreak that occurred in Germany, to confirm the positive status of the animals. The analysed matrices were wild boar serum, blood, bloody swabs, bone marrow, and spleen, as listed in Table S1. Most samples originated from carcasses and were of low quality.

#### 2.1.2. Animal Trial Samples to Represent Different Genome Loads

A second part of the samples (*n* = 87 ASFV-positive) originated from experimentally infected domestic pigs and wild boar at different timepoints post infection with the moderately virulent genotype II ASFV strain “Estonia 2014” [15]. The animal trial, described by Sehl et al. [16], was approved by the competent authority (State Office for Agriculture, Food Safety and Fisheries Mecklenburg Western Pomerania, Rostock, Germany) under reference number 7221.3-2-011/19. This sample set was composed of organ material (lungs, tonsils and spleens) and blood, as listed in Table S1.

#### 2.1.3. African Swine Fever Virus p72 Genotypes Samples

The last part of the samples composing this study (*n* = 39 ASFV-positive) were chosen depending on their p72 genotype. The sample set included culture supernatants, and where available, experimental samples, as detailed below and in Table S1. The origin of these isolates is listed in Table S2.

African ASFV isolates were kindly provided by Dr. Chris Netherton from the Pirbright Institute, Pirbright, Great Britain, with the exception of ASFV Kenya 1033 (genotype IX), which was generously provided by Richard Bishop from the International Livestock Research Institute (ILRI) in Nairobi, Kenya.

To obtain homogeneous sample matrices prior to performing the study, all ASFV isolates were grown for 7 days on peripheral blood mononuclear cells (PBMC) following the protocol described by Pietschmann et al. [17]. Exceptionally, the isolate ASFV “Kenya 1033” [18], which is adapted to WSL-cells, was grown for 5 days on the respective cell line. All samples were stored at −80 °C until further use.

In addition to cell-culture-grown viruses, organs from experimentally infected animals were chosen for this comparative study. These five isolates (“RSA W1/99” (IV); “KAB 6/2” (XI); “MFUE 6/1” (XII), “SUM 14/11” (XIII) and “CHZT 90/1” (XIX)) were intramuscularly inoculated to domestic pigs using 1 × 10^4^ haemadsorbing units per mL. Organ samples, listed in Table S1, were obtained at the endpoint of the trial, this being at 4- to 8-days post inoculation. The animal experiment was approved by the competent authority (State Office for Agriculture, Food Safety and Fisheries Mecklenburg Western Pomerania, Rostock, Germany) under reference number 7221.3-2-011/19.

### 2.2. Viral DNA Extraction

Organ-based samples were first homogenized in 1 mL phosphate-buffered saline with a 5 mm metal bead using a TissueLyser II (Qiagen^®^ GmbH, Hilden, Germany) at 30 Hz for 3 min before extraction was performed on the resulting homogenate supernatant after brief centrifugation.

To assess the general performance of exogenous extraction controls provided by the kit manufacturers, these were added to the lysis buffer upon extraction of the sample panel representing different genotypes (*n* = 39 samples). Preliminary data produced during this study showed that simultaneously adding more than one exogenous control did not impact or interfere with PCR performance (data not shown).

Viral nucleic acids from all samples were extracted using the routine method of the German NRL for ASF, the NucleoMag VET kit (Macherey-Nagel, Düren, Germany) on the automated KingFisher 96 flex platform (Thermo Fisher Scientific, Schwerte, Germany) according to the manufacturer’s recommendations. The study leading to the choice of the extraction method is presented as Supplementary Material (Report S1).

### 2.3. Molecular Assays

The study described here was aimed at comparing the performance of 12 commercially available ASFV real-time PCRs (10 with official certification in Germany) with the OIE-recommended protocol described by Tignon et al. [11]. The Tignon protocol is rather comparable with the commercial kits in terms of amplicon length, probe and protocol design. All qPCR assays targeted slightly different regions of the B464L gene (encoding the p72 capsid protein).

Assay set-up and interpretation of each test results was carried out following the manufacturer’s instructions (as per package information leaflet) or the standard operating procedure of the accredited NRL (SOP LAM04ASP-1).

The kit specifications, including the internal control systems and protocol specifications, are detailed in Table 1.

All PCRs were performed using a C1000^TM^ thermal cycler with the CFX96^TM^ Real-Time System (Bio-Rad, Hercules, CA, USA). Real-time PCR results were recorded as quantification cycle (Cq) values as determined by the CFX Maestro software (Bio-Rad, Hercules, CA, USA).

Genome copy numbers in each sample were calculated in the CFX Maestro software by using a dilution series of ASFV DNA. Briefly, to generate the ASFV standard, DNA from the ASFV isolate “Armenia08” cultured in porcine macrophages was extracted using the QIAamp Viral RNA Mini Kit (Qiagen, Hilden, Germany) according to the manufacturer’s recommendations. Subsequently, the DNA concentration was determined by spectrophotometry using a Nanodrop 2000c (Thermo Fisher Scientific, Lissieu, France) and the exact number of DNA molecules was calculated with an online tool (https://molbiol-tools.ca/; accessed on 1 October 2020). Aliquots were stored at −20 °C and thawed no more than five times.

### 2.4. Data Analysis

Data were recorded and evaluated using Microsoft Excel 2010 (Microsoft Deutschland GmbH, Munich, Germany). One-way ANOVA with Tukey’s post hoc testing was used to compare numerical variables with normal distribution. To determine the agreement between results obtained from two different qPCR systems, i.e., the OIE-recommended assay and a given commercial kit, a comparison of the average differences of genome copies was performed using the Bland–Altman test [19]. The method considers the two sample types to be in agreement if their results fall within the so-called Limit of Agreement (LoA) interval. This interval was calculated using the mean difference ±1.96 standard deviation (SD) of the genome copies obtained using both qPCR systems.

Additionally, diagnostic sensitivity, specificity and precision of each commercial kit were calculated. Briefly, sensitivity was calculated as the percentage of true positives correctly identified in the total number of positive samples, specificity as the percentage of true negatives correctly identified in the total negative samples, and precision as the percentage of true positives correctly identified in the overall true positives and false positives identified [20]. GraphPad Prism 9 (Graphpad Software Inc., San Diego, CA, USA) was used for statistical analyses and graph creation.

## 3. Results

### 3.1. Comparison of Kit Handling and Time Requirements

The required pipetting steps for the commercial kits ranged from two to five with more than two steps for the ViroReal^®^ Kit ASF Virus (5), the Real PCR ASFV DNA Test (4), the INgene q PPA (4), and the VetAlert ASF PCR Test Kit (3). Additional handling steps were mainly related to controls. The volume of template DNA was 5 µL in the vast majority of kits. The Virella ASFV seqc real-time PCR kit requires 6 µL while the Kylt ASF kit and the INgene q PPA require 4 and 2 µL, respectively. The ViroReal^®^ Kit ASF Virus gives a range from 1 to 8 µL but was tested in this study with 5 µL.

With 40 to 45 PCR cycles, estimated run times on the CFX cycler ranged from 1 h 2 min (Virotype ASFV 2.0 PCR kit) to 2 h 18 min (Virella ASFV seqc real-time PCR kit). All but one system had run times of considerably less than 2 h. The five shortest protocols were Virotype ASFV 2.0 PCR kit (1 h 2 min, 40 cyles), ID Gene™ African Swine Fever Triplex (1 h 4 min, 40 cycles), Adiavet ASFV Fast Time (1 h 8 min, 45 cycles), INgene q PPA (1 h 16 min, 45 cycles), and Real PCR ASFV DNA Test (1 h 30 min, 45 cycles).

### 3.2. Evaluation of the Agreement for Viral Genome Detection

A total of 207 selected ASFV samples were analysed in this crosssectional study. Twelve different commercial qPCR methods and one OIE-recommended method as a comparator were performed: Tignon et al. (2011), Virella ASFV seqc real-time PCR kit, VetMax^TM^ ASFV Detection kit, ViroReal^®^ Kit ASF Virus, Kylt ASF, Virotype ASFV PCR kit, Virotype ASFV 2.0 PCR kit, ID Gene^TM^ African Swine Fever Duplex, Real PCR ASFV DNA Test, VetAlert ASF PCR Test Kit, INgene q PPA, Adiavet ASFV Fast Time, and ID Gene^TM^ African Swine Fever Triplex.

**Figure 1:**
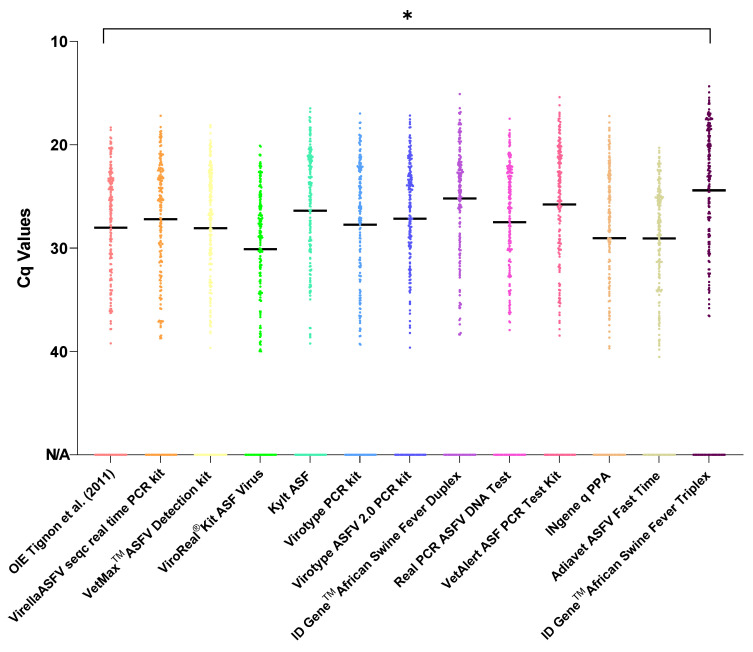
Distribution and variability of Cq values. Nucleic acid extracts from *n* = 207 samples were tested using 13 assays. The line in each plot represents the mean, significance is indicated with an asterisk (*).

Mean Cq values obtained with 11 out of 12 commercial qPCR assays did not differ significantly from the OIE Tignon qPCR method (*p* > 0.05). Nonetheless, statistically significant differences were obtained when comparing the OIE Tignon and ID Gene^TM^ African Swine Fever Triplex (*p* = 0.03). In this latter scenario, a significantly lower Cq value was observed in most of the analysed samples, i.e., deviation for the better (Figure 1).

**Figure 2:**
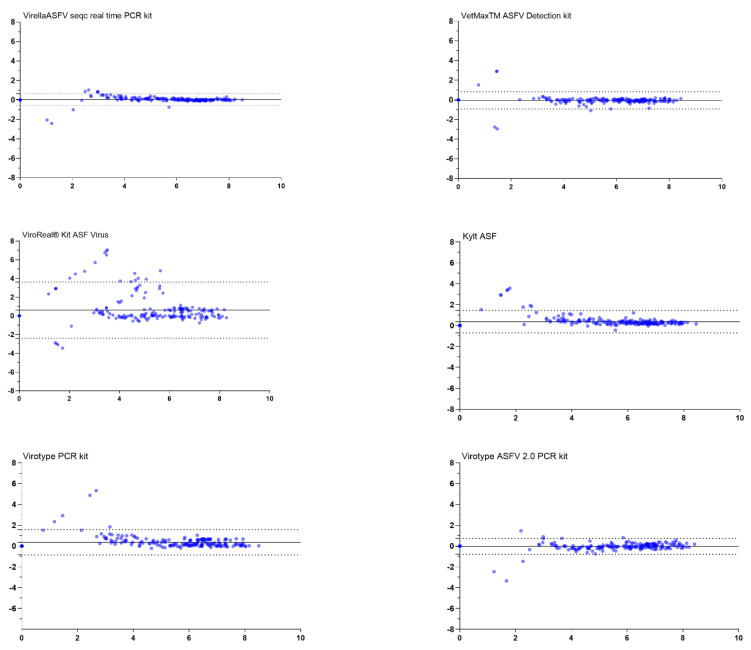

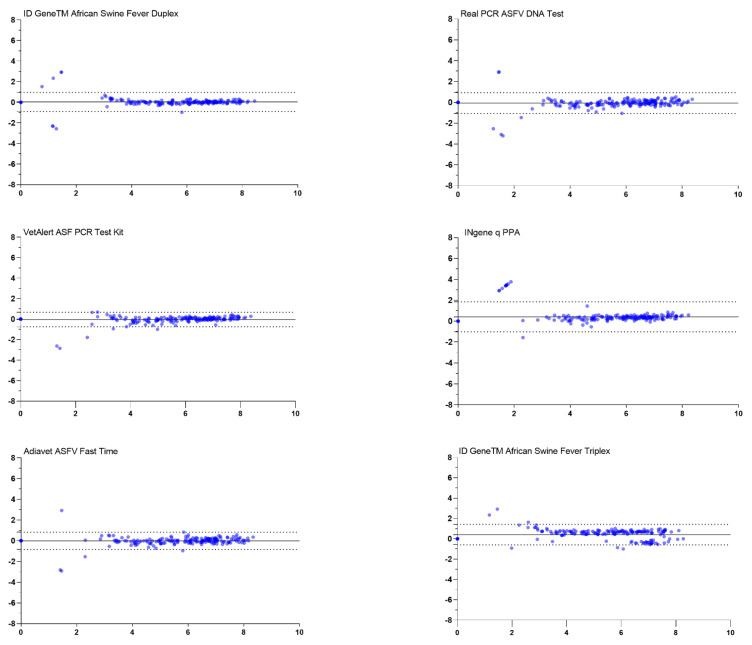
Bland–Altman plots comparing the OIE method designed by Tignon et al. (2011) to commercially available real-time PCR assays. The grey lines represent the bias between the test assays and the OIE method. The grey dotted lines represent the limits (upper and lower) of agreements. The plots (blue dots) show differences between the genome copy numbers inferred from the standard as detected by the OIE method and the tested assays against the average of the genome copy numbers detected.

Further, the agreement between the OIE-recommended method and each of the commercial kits was evaluated using Bland–Altman Plots and point-by-point comparison (see Supplementary Material Figure S1). The bias, i.e., the average discrepancy that could indicate a systematical difference, was highest for the ViroReal^®^ Kit ASF Virus (bias 0.62). This observation coincided with wide limits of agreement and several samples outside the limits (see Figure 2). In this case, the point-by-point comparison showed a high degree of variability with a trend to weaker detections in the ViroReal^®^ Kit, especially with samples of lower genome loads (see Table S1). Manual in-detail comparison revealed that particularly samples of genotype X were detected with a distinct shift in Cq-values and corresponding low genome copy numbers inferred from the standard (see Table S1).

As represented in Figure 2 and Figure S1, all other PCR assays showed that samples ranging from 4 to 8 Log10 genome copies/per run, showing a difference ranging from 0–2 mean ±1.96 SD Log10 genome copies/run, do not exceed the LoA, considering that the two samples are in agreement and the results obtained by any of these PCR assays are comparable, hence being possible to use them interchangeably.

However, a general situation observed for samples below 4 Log_10_ genome copies/run was that both methods were not in agreement, and the real genome copies/run value was under- or over-estimated, depending on the sample, when compared to our comparator (OIE-recommended method). Overall in the tests, samples for which there is a lack of agreement were those with very low genome copies (Figure 2 and Figure S1). It should also be noted that qualitative differences in the results’ interpretations would not have happened for most of these discrepancies.

### 3.3. Evaluation of the Internal Control Systems

All commercial kits combined the detection of the ASF viral genome with at least one internal control system to validate the results, especially if negative. The OIE-recommended assay uses an endogenous internal control (β-actin) that would account for both sample quality and inhibitors of the PCR reaction. Of the commercial PCR kits, five contained an endogenous internal control (ICe), four used an exogenous, i.e., heterologous internal control (ICh), and three offered two internal controls, one endogenous, one exogenous/heterologous (see Table 1).

**Figure 3:**
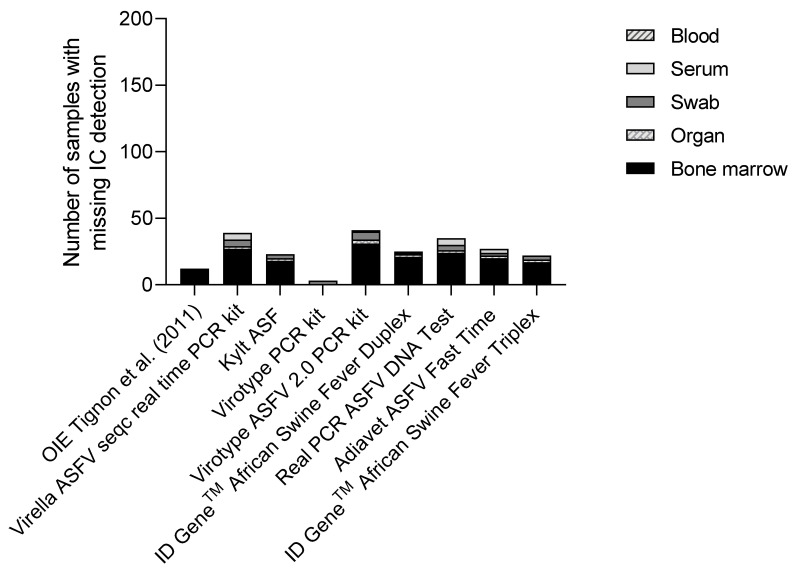
Comparison of endogenous internal control performance across the entire sample set. The overall number of missing internal control detections is split into different sample matrices (the ones that showed the phenomenon), i.e., blood, serum, swab, organ, and bone marrow. It has to be noted that high target amplification (ASFV genome) can lead to missing internal control amplification without impact on the validity of the PCR.

In general, detection of the ICe was successful in over 80% of the analysed samples. The OIE method gave a positive ICe result for 94% of the samples while the commercial kits ranged from 80% (Virotype ASFV 2.0 PCR kit) to 89% (Kylt ASF and ID Gene^TM^ African Swine Fever Triplex). From the overall tested matrices, bone marrow showed the lowest ICe detection, ranging from 10 to 25 samples with missing ICe detection, depending on the qPCR assays, as depicted in Figure 3. Blood- and organ-based samples followed bone marrow in remaining under the established cutoff (Figure 3).

The exogenous controls were only assessed with the sample set representing the different genotypes. On this sample set, the exogenous internal control was detected in all samples, irrespective of genome load and sample matrix.

### 3.4. Sensitivity, Specificity and Precision

Diagnostic performance for all methods is presented in Table 2. Overall, all qPCR assays showed high diagnostic accuracy for detecting ASFV in positive animals and/or cell culture samples. There was no systematic influence by host type or sample matrix. As mentioned above, only one assay (ViroReal^®^ Kit ASF Virus) showed a systemic problem with genotype X, that did, however, not impact the diagnostic outcome in general (mainly a shift in Cq values and inferred genome copy numbers was observed).

For the evaluation of the diagnostic parameters’ sensitivity, specificity and precision, the OIE method was considered together with all other PCRs. The preset status of the sample was taken as standard.

In summary, this study could show that all tests showed high specificity and precision (100%) while sensitivity differed between commercially available assays, ranging from 95% to 100% (see Table 2). According to the highest sensitivity, Virella ASFV seqc real-time PCR kit, VetAlert ASF PCR Test Kit, Virotype ASFV 2.0 PCR kit and, Adiavet ASFV Fast Time were the most accurate tests. It has to be noted, however, that weakly positive samples made the difference and that no repetitions were carried out to test for random effects.

## 4. Discussion

African swine fever is a transboundary and notifiable animal disease of high socioeconomic importance which infects all members of the family *Suidae*. In the absence of a vaccination or treatment option, rapid and reliable diagnostic tools are of paramount importance for the confirmation of clinical cases and thus early implementation of control measures [11]. In this respect, qPCR has become the standard in many countries. Most of the protocols reported so far target a region in the B646L gene (encoding the p72 capsid protein). The present study was conducted at the German NRL for ASF as part of its sovereign duties. In Germany, all tests used for the in vitro detection of notifiable animal diseases have to be certified, and qPCRs must include an internal control. So far, 10 different qPCRs have been certified and at least two more are in the process of licensing. All assays have been approved over time but were never systematically compared.

During this work, all commercial qPCR assays certified by the German Licensing Authority (*n* = 10) and two additional assays were compared with an OIE-recommended qPCR using samples from a wide range of matrices, hosts and genotypes.

In general, almost all assays maintained an easy-handling workflow that can be readily implemented in a routine diagnostic laboratory. Nonetheless, it is remarkable to highlight that kits with ready-to-use reagents, low numbers of pipetting steps or easy opening as well as moderate input volumes maintain human error within acceptable ranges and help avoiding cross-contamination, thus providing more accurate and reliable results in the long run. This was particularly observed by all kits with only two pipetting steps and moderate input volumes (Virella ASFV seqc real-time PCR kit, VetMax™ ASFV Detection kit, Kylt ASF, Virotype ASFV and ASFV 2.0 PCR kit, ID Gene™ African Swine Fever Duplex and Triplex, and Adiavet ASFV Fast Time).

According to the data presented in this study, no host or matrix influence was observed on the ASFV positivity of the qPCR results. Only one kit (ViroReal^®^ Kit ASF Virus) showed a shift in Cq values when detecting the rather exotic genotype X. Overall, differences between the Cq values and inferred genome copy numbers between tested kits were nonsignificant, except for the ID Gene^TM^ African Swine Fever Triplex assay, which showed a tendency of having lower Cq values (higher inferred genome copy numbers) for almost all positively analysed samples.

All samples were extracted using the same protocol, and qPCR performance did not seem to have been influenced by this methodology. This plays as an advantage for the daily broad laboratory routine, since the assays can be integrated into the daily workflow, independently of the extraction method. Nonetheless, it remains to be tested whether the lack of agreement observed in samples with lower genome copies might have relied on the extraction method or could have been prevented using only the extraction methods recommended by the manufacturers. To this end, it would be desirable to perform a follow-up comparison with different extraction protocols recommended by the manufacturers, exceeding the study that is presented in the Supplementary Material.

In addition to the target amplification, the internal control (IC) system of each kit was evaluated. For accredited diagnostic laboratories, robust detection of the internal control is of great importance, as this is the only way to ensure the validity of negative results. In this context, an endogenous internal control, usually a so-called housekeeping gene, is often preferred, as only it allows a statement on the quality of the material used with regard to the presence of host genetic material. Moreover, it does not require addition of a target to all samples and saves hands-on time [21]. Heterologous internal controls are limited to detecting the PCR suitability of the material with respect to inhibitory effects and to basically demonstrating a successful extraction. However, they also work with almost cell-free samples and are not reliant on the expression of the gene [22]. The latter can also be advantageous when dealing with samples of lower quality that are rather often observed with samples from wild boar. From our own experience, however, we have to state that host DNA and thus endogenous internal controls suffer from quality loss (much) earlier than amplification of the target sequence. The commercial PCR kits included in our study had different IC systems. No problems in detection were observed with exogenous controls in the sample set to which all heterologous controls were added in the extraction step. Regarding endogenous internal controls, 8 out of 13 protocols included an endogenous internal control that could be evaluated. On the chosen sample set with limited representation of negative specimens, the internal controls did not fail in a single sample. However, higher variability with up to 20% missing ICe detections was observed in the set of positive samples. It must be noted, though, that the internal controls are limited in favor of target amplification and thus fail when a particularly large amount of viral genome is amplified. Considering this aspect, none of the missing ICe detections would have led to invalidity of the PCR reaction in a diagnostic setting and thus, all systems worked reliably.

When diagnostic key indicators were compared, comparable sensitivity, specificity and precision were determined for all PCR kits, with minor variations. Weak positive samples may generate variability in the chosen approach, i.e., a single diagnostic PCR run, which is random, i.e., had these samples been repeated, they may have been detected in a different kit or failed. That this phenomenon came into play could be inferred by comparing the diagnostic sensitivities of the two virotype kits. Both systems amplify an identical target sequence and run for 40 cycles. It cannot be deduced that the slightly shorter, more complex system should have an actual higher sensitivity. In some cases, the reduced input volume (4 µL vs. 5 µL for the Kylt ASF PCR) or the lower number of PCR cycles (40 vs. 45) might have led to the differences (ID Gene ASF assays). Overall, our results are in line with findings by another NRL that used seven commercially available qPCR kits for their comparison [23].

## 5. Conclusions

Commercial, ideally certified qPCR kits with internal control systems also offer laboratories that are not routinely involved in the establishment and validation of diagnostic protocols the possibility to use reliable and tested systems. We can demonstrate that all systems approved in Germany are comparable to an OIE-recommended PCR assay and provide interchangeable results over a wide range of matrices, genotypes, and genome loads.

Because of the crucial role that laboratory testing plays in the monitoring and guiding of animal health response and the rise of diagnostic setups worldwide, it remains vital to maintain regular monitoring of assays’ performances and comparisons.

## Figures and Tables

**Table 1:** Specifications and qPCR conditions of the used commercial kits for ASFV DNA detection.

Protocol/ PCR Kit.	Internal Control	Volume of DNA Eluates per Test (µL)	Cycles	Estimated Run Time (CFX 96)	Pipetting Steps
OIE Tignon et al. (2011)	Endogenous (β-Actin)	5	45	2 h 25 min *	5
Virella ASFV seqc real-time PCR kit (Gerbion, Kornwestheim, Germany)	Exogenous and endogenous (Succinate-Dehydrogenase)	6	45	2 h 18 min	2
VetMax^TM^ ASFV Detection kit (Thermo Fisher Scientific, Lissieu, France)	Exogenous	5	45	1 h 48 min	2
ViroReal^®^ Kit ASF Virus (Ingenetix, Vienna, Austria)	Exogenous	5 (1–8)	45	1 h 31 min	5
Kylt ASF (AniCon Labor GmbH, Höltinghausen, Germany)	Endogenous (β-Actin)	4	42	1 h 40 min	2
Virotype ASFV PCR kit (Indical, Leipzig, Germany)	Endogenous (β-Actin)	5	40	1 h 43 min	2
Virotype ASFV 2.0 PCR kit (Indical, Leipzig, Germany)	Exogenous and Endogenous (β-Actin)	5	40	1 h 2 min	2
ID Gene^TM^ African Swine Fever Duplex (Innovative Diagnostics, Grabels, France)	Endogenous (housekeeping gene)	5	40	1 h 38 min	2
Real PCR ASFV DNA Test (IDEXX, Hoofddorp, Netherlands)	Endogenous (swine DNA)	5	45	1 h 30 min	4
VetAlert ASF PCR Test Kit (Tetracore, Rockville, USA), not yet approved in Germany	Exogenous	5	45	1 h 36 min	3
INgene q PPA (Ingenasa, Madrid, Spain)	Exogenous	2	45	1 h 16 min	4
Adiavet ASFV Fast Time (Bio-X Diagnostics, Rochefort, Belgium)	Endogenous (RNase P)	5	45	1 h 8 min	2
ID Gene^TM^ African Swine Fever Triplex (Innovative Diagnostics, Grabels, France), not yet approved in Germany	Exogenous and endogenous	5	40	1 h 4 min	2

* depending on the qPCR kit (polymerase) used.

**Table 2:** Sensitivity (Se) and specificity (Sp) of the different kits, calculated from all samples.

Kit Name	Se%	95% CI Min	95% CI Max	Sp%	95% CI Min	95% CI Max
OIE Tignon et al. (2011)	98.32	95.26	99.66	100	87.66	100
Virella ASFV seqc real-time PCR kit	100	97.96	100	100	87.66	100
VetMax^TM^ ASFV Detection kit	98.32	95.26	99.66	100	87.66	100
ViroReal^®^ Kit ASF Virus	99.44	96.94	99.99	100	87.66	100
Kylt ASF	95.53	91.74	98.14	100	87.66	100
Virotype ASFV PCR kit	94.97	91.11	97.79	100	87.66	100
Virotype ASFV 2.0 PCR kit	100	97.96	100	100	87.66	100
ID Gene^TM^ African Swine Fever Duplex	97.21	93.77	99.11	100	87.66	100
Real PCR ASFV DNA Test	98.88	96.07	99.87	100	87.66	100
VetAlert ASF PCR Test Kit	100	97.96	100	100	87.66	100
INgene q PPA	98.32	95.26	99.66	100	87.66	100
Adiavet ASFV Fast Time	100	97.96	100	100	87.66	100
ID Gene^TM^ African Swine Fever Triplex	97.77	94.5	99.4	100	87.66	100
Average	98.16	95.25	99.1	100	87.66	100

## Data Availability

The data that support the findings of this study are available from the corresponding author upon reasonable request.

## Supplementary Materials

The following are available online at https://www.mdpi.com/article/10.3390/v14020220/s1. Table S1: Overview of all samples which were uses in this study with the results of each PCR kit. Table S2: Details of representative ASF viruses used in this study. Report S1: Comparison of different extraction methods. Figure S1: Point-by-point comparison of samples.
